# CCL2 is associated with microglia and macrophage recruitment in chronic traumatic encephalopathy

**DOI:** 10.1186/s12974-020-02036-4

**Published:** 2020-12-05

**Authors:** Jonathan D. Cherry, Gaoyuan Meng, Sarah Daley, Weiming Xia, Sarah Svirsky, Victor E. Alvarez, Raymond Nicks, Morgan Pothast, Hunter Kelley, Bertrand Huber, Yorghos Tripodis, Michael L. Alosco, Jesse Mez, Ann C. McKee, Thor D. Stein

**Affiliations:** 1 Department of Pathology and Laboratory Medicine, Boston University School of Medicine, Boston, MA USA; 2 Department of Neurology, Boston University School of Medicine, Boston, MA USA; 3 Boston University Alzheimer’s Disease and CTE Center, Boston University School of Medicine, Boston, MA USA; 4 VA Boston Healthcare System, Jamaica Plain, 150 S Huntington Ave, Boston, MA 02130 USA; 5 Department of Veterans Affairs Medical Center, Bedford, MA USA; 6 Department of Pharmacology and Experimental Therapeutics, Boston University School of Medicine, Boston, MA USA; 7 National Center for PTSD, VA Boston Healthcare System, Boston, MA USA; 8 Department of Biostatistics, Boston University School of Medicine, Boston, MA USA

**Keywords:** CTE, Chemokine, Neuroinflammation, Tau, Head impacts, TBI, American football, Microglia

## Abstract

**Background:**

Neuroinflammation has been implicated in the pathogenesis of chronic traumatic encephalopathy (CTE), a progressive neurodegenerative disease association with exposure to repetitive head impacts (RHI) received though playing contact sports such as American football. Past work has implicated early and sustained activation of microglia as a potential driver of tau pathology within the frontal cortex in CTE. However, the RHI induced signals required to recruit microglia to areas of damage and pathology are unknown.

**Methods:**

Postmortem brain tissue was obtained from 261 individuals across multiple brain banks. Comparisons were made using cases with CTE, cases with Alzheimer’s disease (AD), and cases with no neurodegenerative disease and lacked exposure to RHI (controls). Recruitment of Iba1+ cells around the CTE perivascular lesion was compared to non-lesion vessels. TMEM119 staining was used to characterize microglia or macrophage involvement. The potent chemoattractant CCL2 was analyzed using frozen tissue from the dorsolateral frontal cortex (DLFC) and the calcarine cortex. Finally, the amounts of hyperphosphorylated tau (pTau) and Aβ_42_ were compared to CCL2 levels to examine possible mechanistic pathways.

**Results:**

An increase in Iba1+ cells was found around blood vessels with perivascular tau pathology compared to non-affected vessels in individuals with RHI. TMEM119 staining revealed the majority of the Iba1+ cells were microglia. CCL2 protein levels in the DLFC were found to correlate with greater years of playing American football, the density of Iba1+ cells, the density of CD68+ cells, and increased CTE severity. When comparing across multiple brain regions, CCL2 increases were more pronounced in the DLFC than the calcarine cortex in cases with RHI but not in AD. When examining the individual contribution of pathogenic proteins to CCL2 changes, pTau correlated with CCL2, independent of age at death and Aβ_42_ in AD and CTE. Although levels of Aβ_42_ were not correlated with CCL2 in cases with CTE, in males in the AD group, Aβ_42_ trended toward an inverse relationship with CCL2 suggesting possible gender associations.

**Conclusion:**

Overall, CCL2 is implicated in the pathways recruiting microglia and the development of pTau pathology after exposure to RHI, and may represent a future therapeutic target in CTE.

## Background

Immune cell trafficking, gliosis, and neuroinflammation are fundamental immune responses designed to protect the brain from harm [1]. Uncontrolled or unregulated neuroinflammation, however, has been implicated as a causative event in many neurodegenerative diseases [2, 3]. One important facet of the inflammatory response is the signaling cascades used to bring inflammatory cells to the areas of damage or pathology (i.e., chemokines). Interestingly, similar inflammatory cell recruitment responses can be observed across distinct injuries. After significant damage to the brain, brain derived microglia and peripheral derived macrophages are recruited to areas of tissue damage in efforts to reduce pathologic protein accumulation and repair the damage [4]. Additionally, microglia recruitment around Aβ plaques in Alzheimer’s disease (AD) are commonly observed. In both CTE and AD, chronic signaling through repetitive injuries or failure to remove toxic protein products is hypothesized to result in constant recruitment of inflammatory microglia/macrophage and may perpetuate a chronic neuroinflammatory response and disease propagation.

Recently, it has been observed that the neuroinflammatory response may be involved in the pathogenesis and disease progression of the neurodegenerative disease chronic traumatic encephalopathy (CTE) [2]. CTE is a progressive tauopathy found in individuals with a history of repetitive head impacts (RHI) typically obtained through playing contact sports such as American football, hockey, soccer, or rugby, in addition to injuries sustained during military service [5, 6]. Evidence from biomechanical computation, helmet sensor data, and neuropathologic autopsy suggests that blood vessels found in the frontal cortex at the depth of the cortical sulcus are observed to be affected the earliest and most severely in CTE, while other regions, including medial and occipital regions such as the calcarine cortex, were relatively spared [6–9]. The amount of neuroinflammation and severity of pathology has been found to be proportional to the time spent playing contact sports and has been suggested to be an important mechanism of pathogenesis [2, 10]. Although it is unclear which specific neuroinflammatory factors are involved, there is strong evidence that microglia are highly involved at all levels of disease severity [2, 11, 12]. Therefore, it would be of interest to better study the signals required to recruit microglia to regions of damage and pathology.

One of the most potent microglia/macrophage chemokines is monocyte chemoattractant protein 1 (MCP1) or more commonly referred to as CCL2 (chemokine (C-C motif) ligand 2) [13]. CCL2 is produced by many CNS resident cells such as astrocytes, neurons, oligodendrocytes, endothelial cells, and also, microglia themselves. Altered expression of CCL2 or its receptor, CCR2, have been found to play mechanistic roles in a variety of brain pathologies. Loss of CCR2 was found to reduce microglia recruitment and increase Aβ in murine models of AD [14]. Elevated levels of CCL2 have been found in acute and chronic multiple sclerosis plaques [15]. Overexpression of CCL2 in experimental stroke models observed increased infarct volume and greater ischemia [16], while CCL2 deficient mice had less tissue damage after permanent middle cerebral artery occlusion [17]. Taken together, CCL2 plays an important role in propagating pathology through recruitment of peripheral and central immune cells to the area of injury and initiates an inflammatory response that is often prolonged and harmful.

Overall, we hypothesize that CCL2 will be positively associated with exposure to RHI and be part of the signaling cascade recruiting microglia to regions of damage and neuropathology in CTE. Herein, we investigate CCL2 protein levels across multiple brain regions to determine if there is a regional specific increase that relates to initial tau deposition. Additionally, we test the hypothesis that although CCL2 might be elevated in other tauopathies such as AD, the regional increased observed in CTE will be distinct. Finally, we explore whether there is a differential effect of pathologic proteins, such as hyperphosphorylated tau or Aβ, on CCL2 expression. The work presented here seeks to identify connections between CCL2 and neuropathology that could become future targets for novel therapeutic strategies aimed to prevent pathology before it begins.

## Methods

### Subjects

Post-mortem human brain tissue was obtained from 261 subjects from different study groups using previously described procedures [18–20]. Different sets of cases were used for histology and immunoassay experiments based on the availability of frozen or formalin-fixed paraffin embedded (FFPE) tissue. Overall, a total of 224 cases with frozen samples were used for the immunoassay experiments and 53 cases with FFPE tissue were used for histology experiments. There were 16 cases that overlapped for both histology and immunoassay analysis and were used for both. Cases for both histology and immunoassay experiments were drawn from multiple brain bank sources. First, 124 individuals that had a history of exposure to American football at either the professional or amateur level were selected from the Understanding Neurological Injury and Traumatic Encephalopathy (UNITE) group consisted of 18 control cases were obtained from the national PTSD brain bank. Control cases lacked a diagnosis of a neurodegenerative disease and did not carry a diagnosis of PTSD. The next group consisted of 119 subjects from clinic and community aging-based brain banks: Boston University Alzheimer’s Disease Center (BU ADC) and Framingham Heart Study (FHS). In the FHS, an athletic history assessment identical to UNITE was performed with the donor’s next of kin [21]. Athletic history was not available for BU ADC participants. In all groups, cases were excluded from the study if they carried a neuropathologic diagnosis of frontotemporal lobe degeneration, neocortical Lewy bodies, or motor neuron disease. A complete description of the neuropathologic analysis is found in the “Neuropathologic examination” section of the methods. Next-of-kin provided written consent for participation and donation. Institutional review board approval for brain donation was obtained through the Boston University Alzheimer’s Disease and CTE center, Human Subjects Institutional Review Board of the Boston University School of Medicine, and Edith Nourse Rogers Memorial Veterans Hospital (Bedford, MA). Demographics, athletic history (type of sports played, level, position, age of first exposure to sports and years playing contact sports), military history (branch, location of service and duration of combat exposure), and traumatic brain injury (TBI) history (including number of concussions) were queried during a telephone interview as detailed previously [22]. Sample data including mean age, gender, and years of playing American football is present in Table 1. A histogram showing the distribution of age of death between all the groups is present in Supplemental figure 1.

**Table 1:** Patient data

	Sample size (*n*)	Sex m/f (% male)	Age at death	PMI (h)	Total years playing American football
**Cases used for immunoassay**					
Non-RHI control	18	14/4 (77%)	47.3 ± 8.6	N/A	N/A
RHI group					
RHI without CTE	20	19/1 (95%)	46.3 ± 20.0	63.7 ± 21.4	8.0 ± 4.2
Low CTE	27	27/0 (100%)	53.7 ± 16.0	51.2 ± 19.0	11.5 ± 6.1
High CTE	47	47/0 (100%)	72.9 ± 11.7	46.0 ± 17.3	16.0 ± 4.4
AD group					
Low AD	60	29/31 (48%)	87.7 ± 8.4	N/A	N/A
Intermediate AD	28	10/18 (35%)	88.8 ± 6.5	N/A	N/A
High AD	24	12/12 (50%)	80.3 ± 10.4	N/A	N//A
**Cases used for histology**					
Non-RHI control	7	4/3 (57%)	70.4 ± 8.1	N/A	N/A
Low CTE	13	13/0 (100%)	43.3 ± 18.5	54.3 ± 61.4	14.2 ± 5.0
High CTE	33	33/0 (100%)	69.5 ± 12.1	57.2 ± 57.6	16.2 ± 4.0

Data expressed as mean ± standard deviation

*AD* Alzheimer’s disease, *CTE* chronic traumatic encephalopathy, *PMI* post-mortem interval, *N/A* not available

### Neuropathologic examination

Pathological processing and evaluation were conducted using previously published methodology [5, 6]. All brain tissue was processed identically by fixation in periodate-lysine-paraformaldehyde and stored at 4 °C. Brain volume and macroscopic features were recorded during initial processing. Twenty-two sections of paraffin-embedded tissue were stained for Luxol fast blue, hematoxylin and eosin, Bielschowsky’s silver, phosphorylated tau (pTau) (AT8), alpha-synuclein, amyloid-β (Aβ), and phosphorylated TDP-43 using methods described previously [23]. A neuropathological diagnosis of CTE was made using the NINDS criteria [6]. Neuropathological criteria for CTE require at least one perivascular pTau lesion consisting of aggregates in neurons, astrocytes, and cell processes around a small vessel; these pathognomonic lesions (referred to as the CTE lesion) are most often distributed at the depths of the sulci in the cerebral cortex and are distinct from the lesions of aging-related tau astrogliopathy by the presence of neuronal tau pathology [24]. Neuropathological evaluation occurred blinded to the clinical evaluation and was reviewed by four neuropathologists (VA, BH, TS, AM); discrepancies in the neuropathological diagnosis were resolved by consensus conference. Cases were only included in the RHI group if they received a negative neuropathologic diagnosis for AD, neocortical Lewy body disease, frontotemporal lobar degeneration, or motor neuron disease. Cases that received a neuropathologic diagnosis of CTE stage 1 or 2 were grouped together as “Low CTE” (*n* = 27) while cases that were CTE stage 3 or 4 were grouped as “High CTE” (*n* = 47). Cases that had a history of playing American football but were not found to have CTE were labeled “RHI without CTE” (*n* = 20). Individuals in the AD cohorts were grouped for AD using the NIA-Reagan criteria [25]. The criteria was as followed: for “High” there needed to be neuritic plaques and neurofibrillary tangles in the neocortex (CERAD frequent, Braak stage of V/VI) (*n* = 24), “Intermediate” required moderate neocortical neuritic plaques and neurofibrillary tangles in limbic regions (CERAD moderate, Braak Stage III/IV) (*n* = 28), “Low” was denoted when there were sparse neuritic plaques and/or neurofibrillary tangles in a more limited distribution and/or severity (CERAD infrequent and/or Braak I/II) (*n* = 60). Cases in the AD group were excluded from the study if they carried a neuropathologic diagnosis of CTE, neocortical Lewy bodies, frontotemporal lobar degermation, or motor neuron disease.

### Immunoassay for CCL2 and Aβ_42_

Flash frozen brain tissue was obtained from the dorsolateral frontal cortex (DLFC) and the calcarine cortex, weighed, and placed on dry ice. Cases were used based on the presence of tissue within the Brain Bank. Not all cases had available calcarine cortex. Freshly prepared, ice cold 5 M guanidine hydrochloride in Tris-buffered saline (20 mM Tris-HCl, 150 mM NaCl, pH 7.4 TBS) containing 1:100 Halt protease inhibitor cocktail (Thermo Scientific) and 1:100 phosphatase inhibitor cocktail 2 & 3 (Sigma) was added to the brain tissue at 5:1 (5 M Guanidine hydrochloride volume (ml): brain wet weight (g)) dilution and homogenized with Qiagen Tissue Lyser LT, at 50 Hz for 5 min. The homogenate was then incubated while rocking overnight at room temperature. Lysate was diluted according to manufacture protocol and spun down at 17,000 g, 4 °C, for 15 min. The supernatant was then applied to Meso Scale Discovery (MSD) Chemokine Panel 1 (human) Kit V-PLEX Plus (Thermo Scientific) and Aβ_42_ ELISA (CAT K15200E-2), following manufactures protocols. Guanidine hydrochloride extraction methods will result in the extraction of both soluble and insoluble forms of proteins for analysis of total levels. Final concentrations were expressed as pg/g.

### Histologic and immunofluorescence staining

Histological staining and analysis of total AT8 pTau density, Iba1+, and CD68+ cell count in the DLFC at the depth of the cortical sulcus was performed using the Aperio ScanScope (Leica) as previously described [26]. For immunofluorescence staining, tissue was extracted from the DLFC, embedded in paraffin, and cut at 20 μm. Immunofluorescence staining was performed using Akoya Bioscience Opal Polaris 7 color manual IHC detection kit as per the manufactures instructions as previously described (tau isoform paper when published). Sections were incubated with antibodies to anti-Iba1 (1:500, Wako), anti-PHF-tau (AT8) (1:1000, Pierce Endogen), anti-TMEM119 (1:100, Abcam), and DAPI. Stained sections were digitized using an Axio Scan.Z1 slide scanner (Zeiss) and visualized using Zen Blue (Zeiss).

### Microglia and macrophage vessel quantitation

Quantification of Iba1+ cell density around CTE lesions as well as microglia vs macrophages density was carried out using Indica Laboratory HALO through manual counts by a blinded observed. To determine the total Iba1+ cell density around CTE lesion blood vessels, three blood vessels that were surrounded by tau and identified as a CTE neuropathologic lesion and three blood vessels not surrounded by tau were selected in each case and used for immunofluorescent analysis. All blood vessels selected were present at the depth of the cortical sulcus in the DLFC. Blood vessels were identified by DAPI stain marking a distinct circular structure and verified with neuropathologists. The number of Iba1+ cells directly contacting the vessel were counted and averaged together.

To determine the abundance of brain derived microglia vs peripherally derived macrophages, dual Iba1 and TMEM119 staining was utilized. As both microglia and macrophages label with Iba1, the presence or absence of TME119 was used to identify cell types [27]. Iba1+ TMEM119+ cells were identified as possible microglia, while Iba1+ TMEM119− cells were identified as possible macrophages. Similar to the total Iba1+ density analysis, blood vessels at the depth of the sulcus in the DLFC were examined to determine if there were changes specific to tau accumulation. Three blood vessels surrounded by tau consistent with a CTE lesion and three blood vessels that lacked tau were selected in each case. The number of Iba1+ TMEM119+ and Iba1+ TMEM119− cells that directly contacted the vessel were counted and averaged together. The number of TMEM119+ and TMEM119− cells were divided by the total Iba1+ cell count to establish percentages of each cell type. Percentages of microglia and macrophages were compared around non-tau and tau containing vessels (control vs lesion vessels). Control cases with no tau pathology were also included. As no vessels contain tau in control cases, only non-tau associated vessels were examined.

### Statistics

Statistical analysis was performed using SPSS (v24, IBM) and Prism (v8, Graphpad Software). Separate two-way ANOVAs were used to examine Iba1+ cell density and the microglia/macrophage ratio around CTE lesioned blood vessel compared to blood vessels that lacked perivascular tau in low and high CTE. Shapiro-Wilk testing revealed CCL2, Aβ42, and AT8 tau density did not have a normal distribution. Since linear regression analysis requires normally distributed data, CCL2, Aβ_42_, and AT8 tau density were transformed using a rank-based method as previously described [28–30]. Briefly, the technique transforms the non-normal variable into a percentile rank for each value, then applies the inverse-normal transformation to the ranks to form a variable which consists of normally distributed Z scores. Further Shapiro-Wilk testing demonstrated transformed values had a normal distribution and were sufficient for linear regression analysis. Separate linear regression analyses were run to compare CCL2 in the DLFC to the total years of playing American football, Iba1+ cell density, and CD68+ cell density. One-way ANOVAs with a Kruskal-Wallis post-test was performed to test differences between CCL2 in the DLFC, calcarine cortex, and the DLFC/calcarine cortex ratio. Ordinal regression was used to examine if the DLFC/calcarine cortex ratio increased according to pathologic disease stage. Multiple linear regressions were used to determine which pathologic protein best correlated with CCL2 levels in the DLFC with age at death, Aβ_42_, and AT8 density as independent predictor variables and CCL2 as the dependent variable across the different analysis groups. Sensitivity analyses run separately within male AD cases and female AD cases were also performed.

## Results

### The CTE pathognomonic lesion recruits Iba1+ cells

**Fig. 1:**
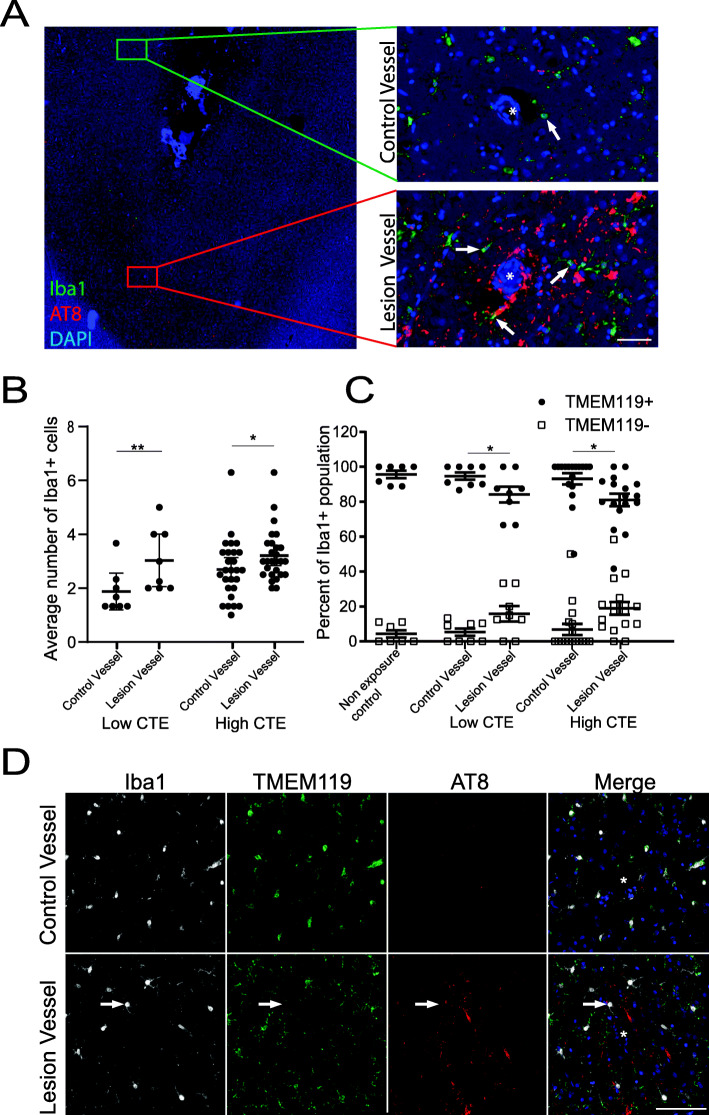
Greater microglia and macrophages are recruited to the CTE lesion blood vessels. The Iba1+ cell density specific to the CTE pathognomonic lesion was investigated to determine if tau specific glial recruitment occurs. **a** Representative image of Iba1+ cells found around control and CTE lesion blood vessels at the depth of the cortical sulcus in the DLFC. Left panel is a low power image of the depth of the cortical sulcus. Right panels are high power images of control and CTE lesion blood vessels. White arrows denote Iba1+ cells with processes contacting blood vessel. Asterisk denotes a blood vessel. Scale bar = 50 μm. **b** Quantitation of the average number of Iba1+ cells found around lesion and control vessels in low and high stage CTE. Each dot represents a single person. **c** Quantitation of the percentage of TMEM119+/Iba1+ and TMEM119−/Iba1+ cells found around the lesion and control vessels in control, mild, and severe CTE. Each dot represents the Iba1+/TMEM119+ (black circles) or Iba1+/TMEM119− (white squares) percentage from a single person. **d** Representative image of Iba1+/TMEM119+ and Iba1+/TMEM119− cells around lesion and control vessels. Increased macrophage recruitment was observed around lesion vessels. Asterisk denotes a blood vessel. Scale bar = 100 μm. Error bars are expressed as mean ± SEM. Statistics between mild and severe CTE generated with a two-way ANOVA. *p < 0.05, **p < 0.01

To determine if the pathology found in CTE was directly related to increased glial cell recruitment, the Iba1+ cell density directly around blood vessels that presented with pTau pathology (i.e., the CTE pathognomonic lesion) was compared to neighboring blood vessels in the same tissue without pTau pathology (i.e., control vessels) (Fig. 1). Overall, an increase in Iba1+ cells was observed around lesion vessels compared to control vessels in the same individual (Fig. 1a, b). Increases were observed in both low and high stage CTE. To determine if the increase in Iba1+ cells was the result of CNS derived microglia or infiltration of peripheral macrophages, dual Iba1/TMEM119 staining and quantitation was performed. TMEM119 has been previously found to only label CNS derived microglia and not peripheral macrophages [27]. When counting the number of Iba1+/TMEM119+ and Iba1+/TMEM119−, it was observed that the majority of cells around all the blood vessels in control, low, and high stage CTE cases were TMEM119+ (Fig. 1c, d). However, a significant increase in TMEM119− cells was found around the lesion vessel in both low and high stage CTE, where they accounted for 15.8% and 19.0% respectively of the total Iba1+ cells when compared to control vessels that had an average of 5.3% and 6.8% respectively (Fig. 1c).

### Glial recruitment signals correlate with exposure to American football, Iba1+, and CD68+ cell density

**Fig. 2:**
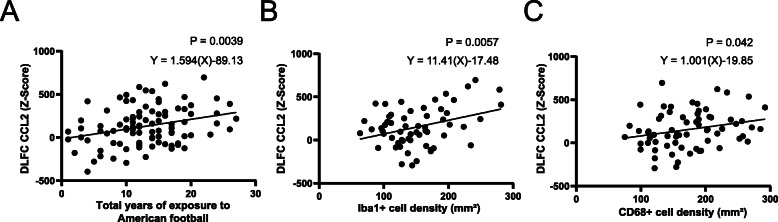
CCL2 levels correlate with the years spent playing American football, the number of Iba1+ and CD68+ cells in cases with RHI. Levels of DLFC CCL2 were compared against **a** the number of years spent playing American football, **b** the density of Iba1+ microglia/macrophages, **c** and the density of CD68+ inflammatory cells found in the DLFC at the depth of the cortical sulcus. All cases had a history of playing American football. Each dot represents a single person. Significance and slope of the line was calculated using linear regression analysis. As CCL2 was found to have a non-normal distribution, a rank bank transformation technique was used to achieve the required normal distribution needed for linear regression analysis. The transformation resulted in normally distributed Z scores which are plotted on the *y* axis

The mechanism behind the Iba1+ recruitment was investigated next. CCL2 is a potent glial chemokine that that is upregulated after impacts, potentially linking RHI to glial recruitment. Analysis of the number of years playing American football (a correlate to the amount of total RHI received) demonstrated a significant correlation between playing longer and increased protein levels of CCL2 within the DLFC (Fig. 2a). Furthermore, CCL2 levels significantly correlated with the overall Iba1+ cell density (Fig. 2b) and CD68+ inflammatory cell density (Fig. 2c). When examining the slops of each linear regression present in Fig. 2a-c, Iba1 density was found to have a larger slope (*Y* = 11.41(*X*)−17.48) when compared to the number of years playing American football (*Y* = 1.594(*X*)−89.13) and CD68 density (*Y* = 1.001(*X*)−19.85). To rule out age as confounding factor, a multiple linear regression model demonstrated that CCL2 significantly correlated with the Iba1+ cell density (*β* = 1.466, *p* = 0.014), but not CD68 cell density (*β* = 0.035, *p* = 0.951), independent of age at death (*β* = 2.955, *p* = 0.061).

### CCL2 is elevated with disease severity

**Fig. 3:**
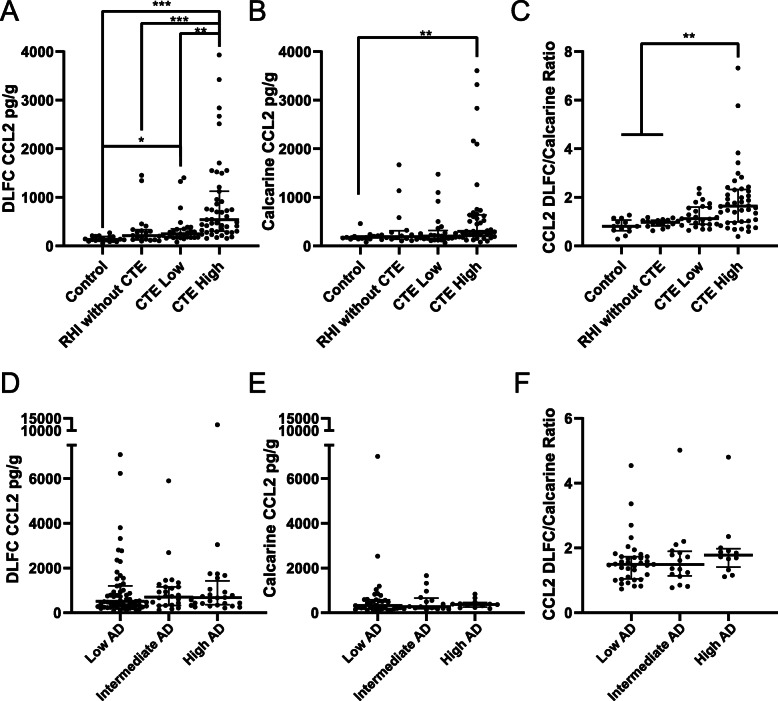
CCL2 is elevated in CTE. Quantitative measurement of CCL2 protein levels in CTE **a-c** and AD **d-f** from the **a**, **d** dorsolateral frontal cortex (DLFC) (control *n* = 18, RHI without CTE *n* = 20, low CTE *n* = 27, high CTE *n* = 47, low AD *n* = 60, intermediate AD *n* = 28, high AD *n* = 24) and **b**, **e** calcarine cortex (control *n* = 13, RHI without CTE *n* = 16, low CTE *n* = 25, high CTE *n* = 42, low AD *n* = 34, intermediate AD *n* = 12, high AD *n* = 19). **c**, **f** To determine if CCL2 was specifically elevated in the DLFC, CCL2 in the DLFC was divided by CCL2 values in the calcarine cortex to obtain a ratio (control *n* = 13, RHI without CTE *n* = 16, low CTE *n* = 25, high CTE *n* = 42, low AD *n* = 37, intermediate AD *n* = 16, high AD *n* = 12). Values over 1 represent more CCL2 in the DLFC compared to the CC. Statistics were generated via a one-way ANOVA with a Kruskal-Wallis post-test comparing differences to the control cases. Each dot represents a single case. Error bars show median and interquartile range. *p < 0.05, **p < 0.01, ***p < 0.001 relative to control cases

To further examine if CCL2 levels were related to neuropathology, CCL2 protein levels were investigated across CTE stages and through multiple brain regions. In the DLFC, a brain region where CTE pathology can be observed early in disease, CCL2 was found to be increased in both low and high stage CTE when compared to control cases (Fig. 3a). When looking at the calcarine cortex, a brain region that is relatively spared from CTE pathology in all but the most severe cases, only differences between control and high stage CTE were observed (Fig. 3b). In order to determine if the observed CCL2 changes were representative of a brain wide increase of CCL2 or a region-specific increase, CCL2 in the DLFC was standardized to CCL2 levels in the calcarine cortex. When comparing across disease groups using an ANOVA, only high stage CTE had a significantly elevated DLFC/calcarine ratio when compared to control cases (Fig. 3c). However, adjusting for age using an ordinal retrogression analysis, the CCL2 ratio was observed to correlate with the step-by-step increase across control, RHI without CTE, low CTE, and high CTE (estimate = 1.622, *p* < 0.001), independent of age at death (estimate = 0.055, *p* < 0.001).

We next wanted to compare CCL2 levels and the differential brain region response, to a similar neurodegenerative disease, Alzheimer’s disease (AD). Using the NIA-Reagan criteria for AD, no significant difference was observed in the DLFC (Fig. 3d) or the calcarine cortex (Fig. 3e). When standardizing DLFC values to calcarine cortex values, no overall difference was observed (Fig. 3f).

### Tau, not Aβ, is best correlated with CCL2 expression

Although AD and CTE are related neurodegenerative diseases that can present with both pTau and Aβ pathology, the pathologic protein most commonly believed to drive disease (pTau for CTE and Aβ for AD) differs. This presents an opportunity to examine differential effects of various pathologic proteins on CCL2 levels and explore possible novel interaction pathways. Multiple linear regression analysis was performed to determine if pTau or Aβ was related to CCL2 levels in the DLFC. Overall, the amount of pTau, as measured by AT8 staining, was significantly correlated with CCL2 independent of age at death in both CTE and AD (Table 2). Aβ_42_ levels, measured by immunoassay, were found to not correlate with CCL2 when grouping all cases together. However, when segregating male and female AD cases, a trend toward a negative correlation between Aβ_42_ and CCL2 in men was observed (Table 3).

**Table 2:** Multiple linear regression model demonstrating AT8 is the strongest correlate of DLFC CCL2 levels

	All			Control			CTE			AD		
	*β*	SE	*p* value	*β*	SE	*p* value	*β*	SE	*p* value	*β*	SE	*p* value
Age at death	0.239	1.015	**0.003**	−0.109	8.784	0.771	−0.030	1.536	0.810	−0.068	2.821	0.539
Aβ_42_	0.013	0.022	0.864	0.221	0.109	0.574	0.052	0.029	0.626	−0.081	0.035	0.437
AT8 density	0.408	< 0.001	**< 0.001**	0.295	0.001	0.435	0.592	< 0.001	**< 0.001**	0.304	< 0.001	**0.007**

*β* standardized beta, *SE* standard error, *CTE* chronic traumatic encephalopathy, *AD* Alzheimer’s disease

**Table 3:** Aβ_42_ appears to have differential effects with CCL2 in males and females in the AD group

	Male			Female		
	*β*	SE	*p* value	*β*	SE	*p* value
Age at Death	−0.221	3.587	0.142	0.116	4.262	0.481
Aβ_42_	−0.240	0.042	0.086	0.121	0.059	0.440
AT8 Density	0.277	< 0.001	0.068	0.349	< 0.001	**0.038**

*β* standardized beta, *SE* standard error

## Discussion

Here, we have shown that there is increased Iba1+ cell recruitment around pTau containing blood vessels in CTE. When investigating possible recruitment factors, the chemokine CCL2, was observed to correlate with years of playing American football, number of Iba1+ cells, and number of CD68+ neuroinflammatory cells. Further analysis demonstrated protein levels of CCL2 were elevated preferentially in the frontal cortex, a region where CTE pathology can first be observed. CCL2 did not correlate with the NIA-Reagan criteria for AD likelihood in a separate group of cases that lacked a significant history of exposure to head impacts. Analysis of the specific effect of pTau and Aβ_42_ demonstrated that pTau correlated with CCL2 in both AD and CTE cases. Aβ_42_ did not have any correlation on CCL2 in CTE cases; however, there was a negative correlation found between Aβ_42_ and CCL2 in AD males. Overall, the present study expands on previous work demonstrating neuroinflammation and glial recruitment is a consequence of RHI and might be implicated in CTE pathogenesis [2].

The current results suggest CCL2 is part of the neuroinflammatory signaling cascade after RHI. The initial mechanisms behind CCL2 elevation after head impacts may be protective. Recruiting microglia and monocytes to areas of damage is critical to remove dead tissue, prevent infection, and promote recovery. However, prolonged, chronic, or intense signaling turns the initial protective response into a damaging one. Consistent with chronic signaling through RHI, CCL2 was found to be correlated with the years spent playing American football. Additionally, CCL2 trended higher in the RHI without CTE group compared to controls suggesting the chronic exposure and the damage associated with playing American football is potentially sufficient to induce CCL2 in the brain, independent of pTau. After injury, brain derived microglia and peripheral-derived macrophages are commonly observed to be recruited to the region of damage. In CTE, the area of most concentrated RHI damage are blood vessels at the depths of the cortical sulcus in the frontal cortex [6]. In agreement, elevated Iba1+ cells were seen to accumulate in correlation with perivascular deposits of pTau (i.e., the CTE pathognomonic lesion [6]). Although the majority of accumulating cells were Iba1+/TMEM119+, a subset were TMEM119− and believed to be infiltrating peripheral macrophages, which has been previously reported after RHI [8]. Future work will be needed to verify the peripheral macrophages involvement, as it is possible the TMEM119− population are microglia that downregulate the TMEM119 gene expression during inflammation. Although CCL2 was observed to correlate with both increased number of microglia and increased inflammatory activity, it is likely that CCL2 is only involved in the recruitment of cell and the glial inflammatory response occurs via secondary factors (i.e., proximity to damage neurons, pTau, or Aβ). To that end, multiple linear regression modeling demonstrated that when including Iba1, CD68, and age at death into the same model, only the Iba1+ cell density correlated with CCL2. This suggests that in the current study, CCL2 is only recruiting microglia to areas of damage.

Although the results suggest CCL2 and the neuroinflammatory response could be elevated prior to pTau accumulation, it is difficult to determine causality and the order of events due to the cross-sectional nature of studies using postmortem human tissue. However, regardless of which occurs first, our previous work suggests that once there is an increase in neuroinflammatory microglia, a feedback loop occurs where pTau causes inflammation which further induces pTau deposition [2]. In addition to enhanced pTau deposition, the inflammatory response results in tissue damage [31]. In experimental models, loss of CCR2 has been observed to block recruitment and reduce the area damaged suggesting a beneficial effect of limiting microglia and macrophages [31]. Moreover, several studies have confirmed that blocking the CCL2 signaling pathway through genetic means or small molecules have potentially protective effects after head impacts [32]. Considering these studies, future research should examine the possible beneficial effect of blocking microglia recruitment and its outcome on local neuroinflammation and pTau deposition.

In addition to blood vessels, the frontal cortex is where CTE pathology is typically first observed and the calcarine cortex is relatively spared [6]. It is not entirely clear why the frontal cortex as opposed to the parietal or temporal cortex exhibits pathology first, but it hypothesized to be related to the physical area of contact (i.e., helmet to helmet hits) in addition to kinetics and physics of head impacts [7, 33]. This would suggest that changes related to CTE pathology would initially be restricted to the region where pathology occurs first. Several studies have shown that CCL2 was elevated in the CSF after TBI [34, 35]. However, tissue regional specificity of CCL2 in the brain has not been examined before. Here, we show that in low and high stage CTE, when standardizing frontal cortex CCL2 values to those in the calcarine cortex, more CCL2 was found in the DLFC. Ordinal regression also demonstrated a step-by-step increase from control cases, RHI without CTE, low CTE, and high CTE when controlling for age of death. This comparison provides compelling evidence that glial recruitment signals are directed to the regions of greatest injury and CTE pathology as opposed to a non-specific TBI-related brain-wide increase.

CCL2 can be produced as a consequence of a variety of stimuli. Although the present study focused on RHI, increases in CCL2 has been found in various neurodegenerative diseases and other injuries. To further explore how similar or different the CCL2 dynamics found after RHI were to other stimuli, subjects with AD without a history of RHI were examined. The inclusion of cases with AD allowed investigation into how factors such as aging or specific pathogenic proteins like pTau and Aβ could factor into CCL2 production independent of head impacts. Using multiple linear regression modeling, it was observed that pTau did correlate with CCL2 in both AD and CTE. Additionally, age was observed to correlate with CCL2 as well, demonstrating exposure to head impacts is not the only driving factor in CCL2 expression. This represents the diverse nature of the immune response with multiple stimuli converging on specific pathways. Neurons and microglia have significant cross-talk with a diverse range of receptors designed to maintain a homeostatic environment [36]. Disruption of this crosstalk will lead to neuroinflammation. Head impacts and the subsequent neuronal damage represent one way to disrupt this normal homeostatic relationship. Additionally, pTau aggregation in neurons also induces neuronal dysfunction and damage, independent of RHI. This shows that although the stimulus is different, similar CCL2 increases are observed across multiple diseases. Although this limits CCL2s ability to be used as a specific biomarker for disease, it does suggest that CCL2 could be a general target for therapeutics that possibly might be effective across multiple disorders.

Surprisingly, Aβ levels were not found to correlate with CCL2. Aβ plaques are associated with a microgliosis and are a consistent feature of AD and variably present in CTE [37]. However, our results demonstrated that CCL2 was significantly associated with pTau and not Aβ_42_ in the overall group as well as in both CTE and AD groups examined separately. It is unclear why the Aβ results in the current study does not agree with reports, mainly using AD transgenic mouse models, suggesting CCL2 facilitates Aβ deposition [38, 39]. One explanation is that many of the Aβ related studies are performed in an in vitro setting or murine models of disease and do not fully recapitulate the human in vivo system. An important distinction is that many transgenic mouse lines only express Aβ or pTau pathology in isolation, when the true human disease environment is much more complex. Similarly, the current study did not examine separate effects of soluble vs insoluble versions of both pTau and Aβ which likely drive different aspects of disease. Additionally, the current study only examines the neurodegenerative environment at the time of death. This snapshot in time does not capture early pathologic changes at the beginning of disease. It is possible early CCL2 activity does drive initial Aβ deposition, however, this effect subsides after several years when pathology is more severe. Interestingly, when separating the AD group by gender, a trend toward an inverse relationship was observed in males. This is consistent with the role microglia play in phagocytosing Aβ [40]. In cases with a higher CCL2 signal, more microglia might be recruited to phagocytose plaques resulting in less Aβ. However, as previously mentioned, it is difficult to fully observe this effect when other pathogenic proteins, head impacts, and age also affect CCL2 levels. Further investigation into the possible unique effects of Aβ will be needed.

The current study is not the first examination into chemokines in CTE. Previous work has demonstrated the chemokine CCL11 was elevated in CTE [26]. Furthermore, CCL11 was able to differentiate CTE and AD. An important distinction is that CCL11 was believed to be produced by the choroid plexus and not locally in areas of damage [41]. Additionally, CCL11 has a broader range of action and can affect more diverse immune cell types, although it can also induce a glial inflammatory response [42–45]. This represents the complex nature and interplay of the immune response and highlights the idea that no chemokine exists in isolation. Therefore, it will be important to examine how other chemokines act in concert with CCL2 and regulate glial recruitment and neuroinflammation in order to get a clear understanding of the neuroinflammatory cascade that occurs after head impacts.

## Conclusion

In conclusion, these results begin to reveal a possible mechanistic pathway for the association of CCL2 and neuroinflammation with CTE. Initially, exposure to RHI leads to tissue damage in the frontal cortex at the depth of the cortical sulcus and around blood vessels. To repair the damage, microglia and macrophages are recruited via targeted CCL2 signaling. After years of sustained RHI and chronic neuroinflammation, pTau deposition begins. The comparison across multiple neurodegenerative diseases suggest that convergent mechanisms between AD and CTE such as pathologic protein deposition and normal aging can also increase CCL2 levels through mechanisms distinct from RHI. It is feasible that the presence of pTau and neuronal dysfunction results in even further CCL2 production and glial recruitment contributing to a proposed vicious circle that might drive CTE pathology. Overall, we suggest that CCL2 might be a possible mechanism of early immune cell recruitment to areas of RHI damage and could be a novel target for future therapeutics to abate or reduce neuroinflammation.

## Supplementary Information

**Additional file 1: Supplemental Figure 1**. Distribution of age at death across sample groups. Histogram showing the distribution of the ages at death for A) Control, B) RHI without CTE, C) Low CTE, D) High CTE, E) Low AD, F) Intermediate AD, and G) High AD. Cases are binned for ever 5 years.

## Data Availability

The datasets used and analyzed during the current study are available from the corresponding author on reasonable request.

## Abbreviations

AD: Alzheimer’s disease
Aβ: Amyloid beta
BU ADC: Boston University Alzheimer’s Disease Center
CCL2: C-C Motif chemokine ligand 2
CNS: Central nervous system
CTE: Chronic traumatic encephalopathy
DLFC: Dorsolateral frontal cortex
FHS: Framingham Heart Study
MCP1: Monocyte Chemoattractant Protein-1
N/A: Not available
NIA: National Institute of Aging
PMI: Post-mortem interval
pTau: Hyperphosphorylated tau
RHI: Repetitive head impacts
SD: Standard deviation
SE: Standard error
TBI: Traumatic brain injury
TMEM119: Transmembrane Protein 1
UNITE: Understanding neurological injury and traumatic encephalopathy

## Supplementary Information

**Supplementary information** accompnies this at 10.1186/s12974-020-02036-4.
