# Supplementary figures and images for: CCL2 is associated with microglia and macrophage recruitment in chronic traumatic encephalopathy
Source: J Neuroinflammation. 2020 Dec 5;17:370. doi: 10.1186/s12974-020-02036-4 (PMC7718711; doi:10.1186/s12974-020-02036-4)

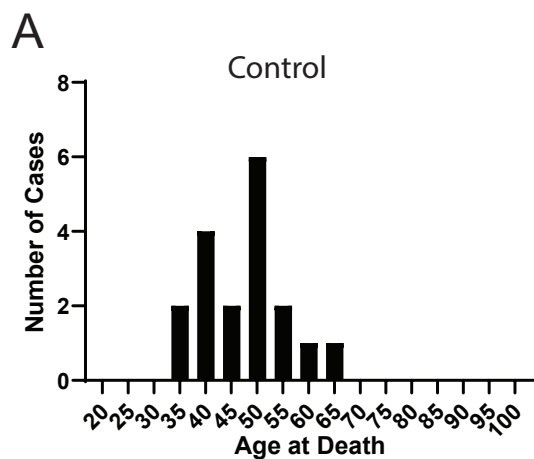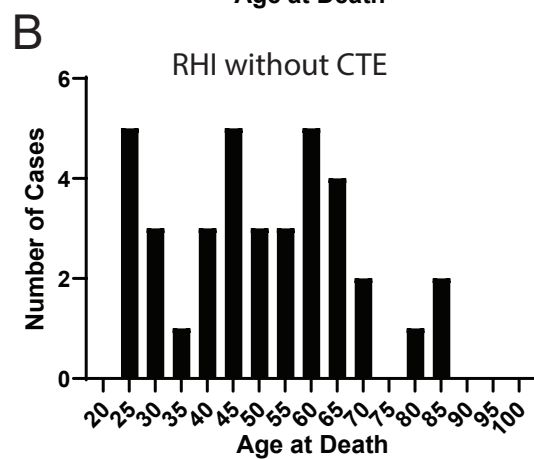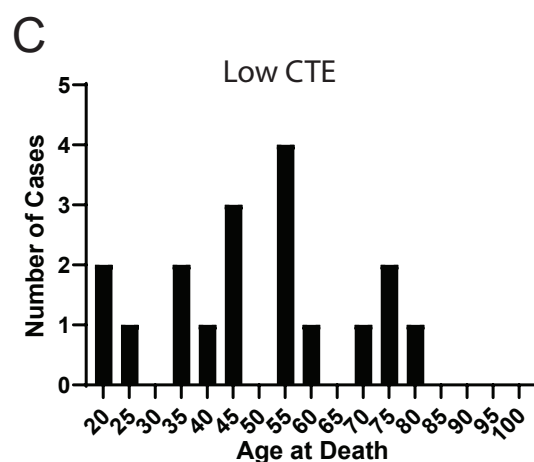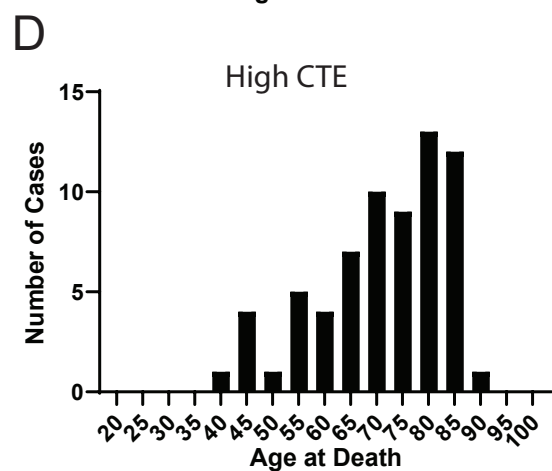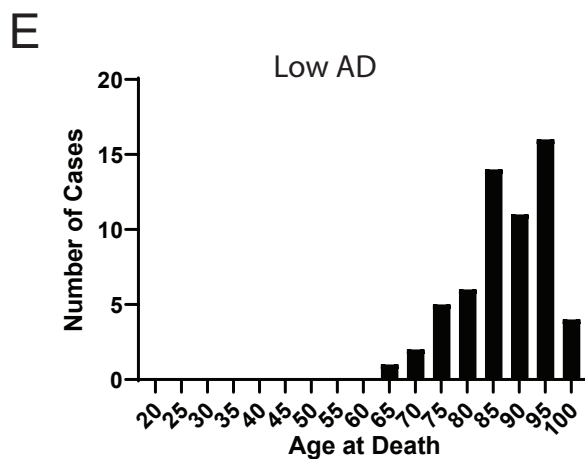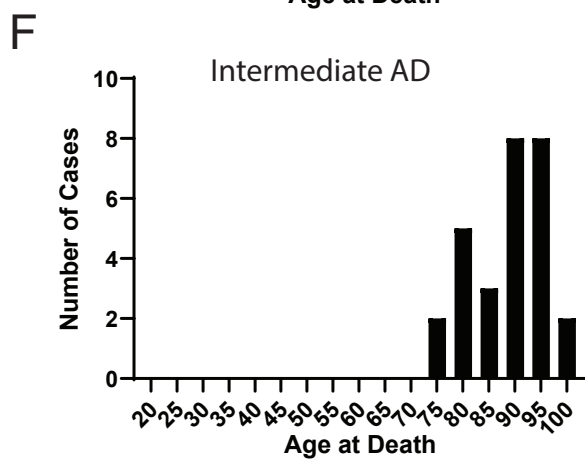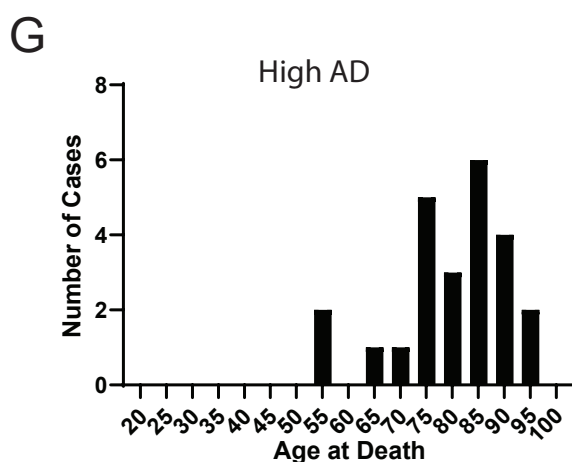

Supplement: Supplementary file 1 — Additional file 1: Supplemental Figure 1. Distribution of age at death across sample groups. Histogram showing the distribution of the ages at death for A) Control, B) RHI without CTE, C) Low CTE, D) High CTE, E) Low AD, F) Intermediate AD, and G) High AD. Cases are binned for ever 5 years. [file 12974_2020_2036_MOESM1_ESM.pdf]
